# Genome-Wide Identification and Characterization of the Vacuolar H^+^-ATPase Subunit H Gene Family in Crop Plants

**DOI:** 10.3390/ijms20205125

**Published:** 2019-10-16

**Authors:** Chen Kang, Fengjie Sun, Lei Yan, Rui Li, Jianrong Bai, Gustavo Caetano-Anollés

**Affiliations:** 1 College of Biology Engineering, Shanxi University, Taiyuan 030006, Shanxi, China; kangchen@sxagri.ac.cn; 2 Institute of Crop Sciences, Shanxi Academy of Agricultural Sciences, Taiyuan 030031, Shanxi, China; yanlei@sxagri.ac.cn (L.Y.); lirui@sxagri.ac.cn (R.L.); 3 School of Science and Technology, Georgia Gwinnett College, Lawrenceville, GA 30043, USA; fsun@ggc.edu; 4 Department of Crop Sciences, University of Illinois at Urbana-Champaign, Urbana, IL 61801, USA

**Keywords:** V-ATPase subunit H, exon-intron structure, alternative splicing, structural domains, vacuole

## Abstract

The vacuolar H^+^-ATPase (V-ATPase) plays many important roles in cell growth and in response to stresses in plants. The V-ATPase subunit H (VHA-H) is required to form a stable and active V-ATPase. Genome-wide analyses of *VHA-H* genes in crops contribute significantly to a systematic understanding of their functions. A total of 22 *VHA-H* genes were identified from 11 plants representing major crops including cotton, rice, millet, sorghum, rapeseed, maize, wheat, soybean, barley, potato, and beet. All of these *VHA-H* genes shared exon-intron structures similar to those of *Arabidopsis thaliana*. The C-terminal domain of VHA-H was shorter and more conserved than the N-terminal domain. The *VHA-H* gene was effectively used as a genetic marker to infer the phylogenetic relationships among plants, which were congruent with currently accepted taxonomic groupings. The *VHA-H* genes from six species of crops (*Gossypium raimondii*, *Brassica napus*, *Glycine max*, *Solanum tuberosum*, *Triticum aestivum*, and *Zea mays*) showed high gene structural diversity. This resulted from the gains and losses of introns. Seven *VHA-H* genes in six species of crops (*Gossypium raimondii*, *Hordeum vulgare*, *Solanum tuberosum*, *Setaria italica*, *Triticum aestivum*, and *Zea mays*) contained multiple transcript isoforms arising from alternative splicing. The study of cis-acting elements of gene promoters and RNA-seq gene expression patterns confirms the role of *VHA-H* genes as eco-enzymes. The gene structural diversity and proteomic diversity of *VHA-H* genes in our crop sampling facilitate understanding of their functional diversity, including stress responses and traits important for crop improvement.

## 1. Introduction

The large central vacuole is one of the most distinctive organelles that are essential for plant viability [1]. Central vacuoles function as reservoirs for ions and metabolites, which allow buffering of changes in nutrient and toxic components, including toxic metals and excessive salts that plants frequently encounter. More importantly, the vacuoles are necessary for plant growth and development. In fact, the expansion of a plant cell is achieved by osmotically driving water influx into the vacuole that, in combination with the cell wall, provides the driving force for cell growth and reversible vacuolar volume changes. The vacuoles also enable plants to detoxify the cytosol and serve as lysosome-like organelles that digest the endocytic and autophagic cargoes [2].

All of the vacuolar functions require massive fluxes of molecules across the tonoplasts, which are assumed to be energized by the proton gradient catalyzed by the vacuolar H^+^-ATPase (V-ATPase) functioning as an electrogenic H^+^ pump [3]. The V-ATPase is a membrane-bound transport protein located at the tonoplast and various components of the endomembrane system, including the endoplasmic reticulum, Golgi apparatus, lysosomes, and secretory vesicles [3]. The V-ATPase is responsible for the acidification of intracellular organelles in all eukaryotic cells, which is induced during a variety of cellular processes [4,5,6]. For example, the V-ATPase hydrolyzes ATP to pump protons from the cytosol into various organelles, including vacuoles, endosomes, and the Golgi apparatus [7]. The plasma membrane H^+^-ATPase extrudes H^+^ from the cell and energizes the uptake and release of many types of nutrients across the plasma membranes of plant cells. Since it is also involved in eco-physiological adaption at the molecular level, the V-ATPase is considered an eco-enzyme in plants [8]. On the one hand, the V-ATPase functions as a house-keeping enzyme to maintain the homeostasis of both cytosolic ions and cellular metabolisms. On the other hand, under environmental stress, the V-ATPase functions as a stress-responsive enzyme that undergoes moderate changes in expression of subunits and modulations of enzymatic structures [9,10]. To summarize, the H^+^ pump (i.e., the V-ATPase) plays important roles in unique physiological processes of plants, including nutrient transport, flowering, stress tolerance, and the particular functions of guard cells and the vascular and meristematic tissues [11,12].

The V-ATPases are present not only in plant cells [13], but also in cells of other eukaryotes, including fungi, insects, and mammals [14,15,16]. The V-ATPases are highly abundant in the tonoplast, making up 6.5%–35% of the total proteins in the tonoplast of a plant cell [17,18]. Structurally, the V-ATPase is composed of two distinct multi-subunit, functional complexes: A membrane integral domain (V_o_) and a membrane peripheral domain (V_1_) [19]. The V_o_ consists of six subunits (a, c, c′, c″, d, and e) in the tonoplast and belongs to the proton-conducting region. The V_1_ is made up of eight subunits (A, B, C, D, E, F, G, and H) functioning in hydrolysis and regulations [19], with subunits A to G responsible for assembly of the entire V-ATPase [20,21], while H is the only subunit that is not required for assembly of the V-ATPase [21]. In yeast, the V_1_–V_o_ complexes of V-ATPase lacking subunit H are not active with reduced stability, suggesting that the subunit H is an activator of the fully assembled V-ATPase complex [22,23].

The crystal structure with high resolution of subunit H of the V-ATPase was first solved in yeast [24], revealing that the protein of subunit H is mainly α-helical and contains two domains, the N-terminal domain (348 amino acids at positions 1–348) and the C-terminal domain with (126 amino acids at positions 353–478). The N-terminal and C-terminal domains are connected by a linker region of four amino acids at positions 349–352. The N-terminal domain is sufficient to activate the ATP hydrolysis activity of the V-ATPase complex, while the C-terminal domain is required for the ATPase activity coupled to proton translocation [25]. It has been demonstrated that the first 179 amino acids of the N-terminal domain are not required for the activation of the complete functions of the V-ATPase complex, while the minimal region of subunit H capable of activating the V-ATPase contains 174 amino acids at positions 180–353 of the N-terminal domain [26].

The V-ATPase plays many key roles in plant growth and development and in stress response, while subunit H is vital to the activity and stability of V-ATPase. Studies have shown that overexpression of three genes in *Suaeda corniculata* (*ScVHA-B*, *ScVHA-C*, and *ScVHA-H*) coding for 3 subunits of V-ATPase improves the tolerance in transgenic alfalfa to salt and saline-alkali stresses [27]. However, little is known about the functions of subunits H in main crops, which are generally the major sources of food and renewable energy. Here we identified the genes encoding the subunit H of V-ATPase in 11 major crops, cotton, rice, millet, sorghum, rapeseed, maize, wheat, soybean, barley, potato, and beet. We further studied the gene structures, proteomic structures, and splice variants of the subunit H in the crops using various bioinformatic, phylogenetic, and protein motif analyses. The results broaden our understanding of the roles of subunit H in plants and provide a framework for further functional investigations of subunit H encoding genes for crop improvement.

## 2. Results

### 2.1. Identification of VHA-H Gene Family Members in the Main Crops

A total of 22 *VHA-H* genes containing typical V-ATPase-H N-terminal and V-ATPase-H C-terminal domains were obtained from 11 major crops following definitions of the Pfam database (Table 1). A single *VHA-H* gene was identified in each one of the five species (*Oryza sativa*, *Beta vulgaris, Hordeum vulgare, Setaria italica*, and *Sorghum bicolor*), while 2, 6, 2, 2, 3, and 2 *VHA-H* genes were revealed in *Gossypium raimondii*, *Brassica napus, Glycine max*, *Solanum tuberosum*, *Triticum aestivum*, and *Zea mays*, respectively. The lengths of ORFs of these 22 genes ranged from 1347 bp (*Glycine max*) to 1488 bp (*Oryza sativa*), while the numbers of exons were 11 (17 genes in 8 species), 12 (one gene in 4 species including *Gossypium raimondii*, *Oryza sativa*, *Setaria italica*, and *Sorghum bicolor*), or 13 (one gene in *Brassica napus*). Analysis of the protein sequences and their physical and chemical properties revealed that the lengths of the deduced polypeptides ranged from 448 (*Glycine max*) to 495 amino acids (*Oryza sativa*), and the PI values of proteins were mostly around 6−7 except for one rice gene (*OsiVHA-H*), which had the highest PI value of 9.07.

### 2.2. Protein Sequence Alignments and Phylogenetic and Motif Analyses

**Figure 1:**
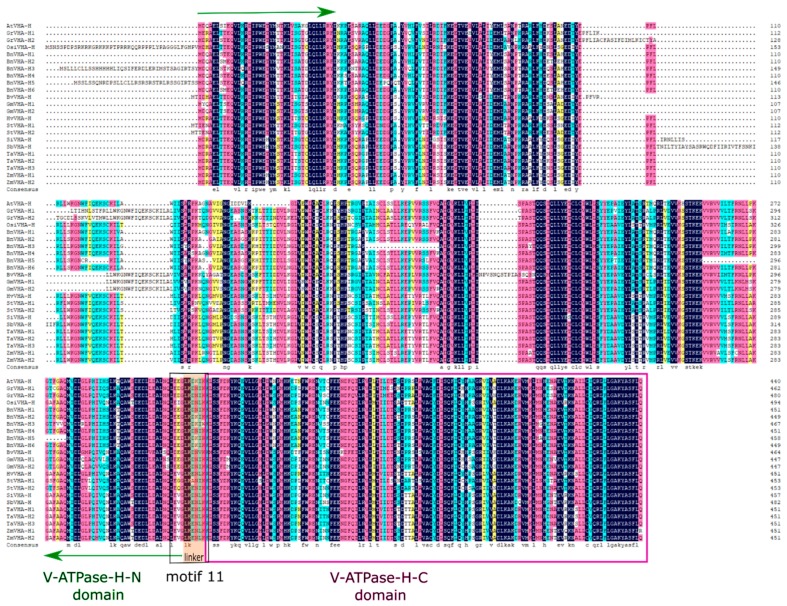
Alignment of the putative amino acid sequences of 23 *VHA-H* genes in 12 species of plants. The N-terminal domain is located between two green arrows. The C-terminal domain is indicated inside a pink frame. The linker region of six amino acids highlighted with the orange background is a portion of motif 11, which is inside a black frame.

**Figure 2:**
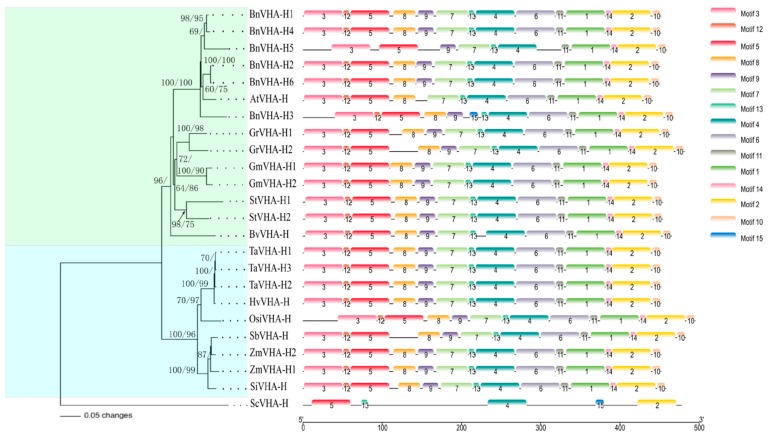
The neighbor-joining tree and motif compositions of the 24 *VHA-H* genes from 12 species of plants and yeast (*Saccharomyces cerevisiae*, *ScVHA-H*), which was used as outgroup. The green and blue backgrounds on the tree indicate the clades of dicots and monocots, respectively. Bootstrap values based on 1,000 replicate analysis and the values of the posterior probability based on Bayesian analysis separated by forward slashes are given on the branches. The bar on the bottom left indicates 0.05 base differences per amino acid position. A total of 15 motifs represented by color bars are revealed on the 24 *VHA-H* genes. The ruler on the bottom right displays the location information of each motif on the amino acid chain.

**Figure 3:**
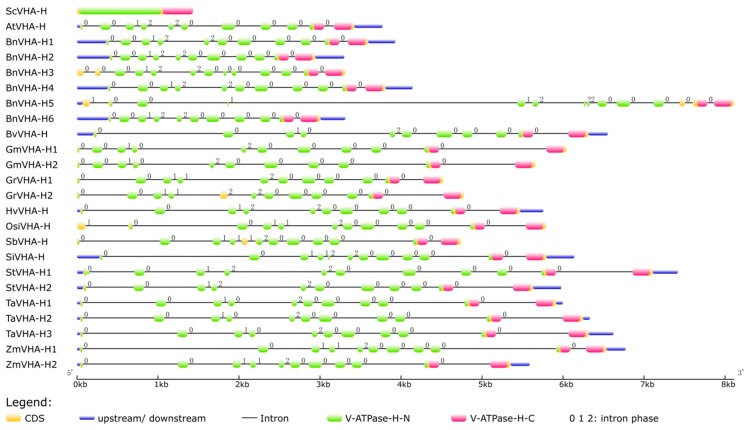
Exon-intron structures of the 24 *VHA-H* genes in 12 species of plants and yeast. Exons and introns are illustrated with filled boxes and single black lines, respectively. Conserved protein sequences of V-ATPase-H N-terminal and C-terminal domains are marked in green and pink, respectively, while the rest of the CDS is highlighted in yellow. Untranslated regions (UTRs) are displayed using blue rectangles at both ends of the sequences. Intron phases 0, 1, and 2 are shown on the top left of the black lines. The ruler at the bottom indicates the lengths of the genes in kb.

Aligned protein sequences of the identified *VHA-H* genes revealed they were highly conserved, with the C-terminal domain being shorter and more conserved than the N-terminal domain (Figure 1). The linker between the two domains in all of the identified *VHA-H* genes contained six amino acids in length. A total of 15 conserved motifs of the protein sequences encoded by the *VHA-H* genes were revealed using MEME. The amino acid compositions of these motifs are presented in Figure 2. These conserved motifs encoded by the *VHA-H* genes in most of the main crops (except for *BnVHA-H3* and *BnVHA-H5* of *Brassica napus*) were highly consistent in types, numbers, and distribution modes (Figure 2). They shared the same arrangement pattern of motifs 1–14. However, *BnVHA-H3* did not contain motif 7, which was replaced with motif 15 at this position, while *BnVHA-H5* lacked motifs 6, 8, and 12. These motif arrangements of these two genes all occurred in the N-terminal domain (Figure 2). Motif 11 consisted of 11 amino acids, including a linker region of six amino acids in length (Figure 1, Figure 2). The E-values of motifs 1 and 2 were the highest among all of the 15 motifs and the C-terminal domain was composed mainly of both motifs 1 and 2, supporting again the C-terminal domain being more evolutionarily conserved than the N-terminal domain (Table 2, Figure 2). Overall, the positional changes of the motifs of all of the identified genes were consistent within the positions of both the N-terminal and the C-terminal domains (Figure 3).

The phylogenetic tree showed that the 23 protein sequences were clustered into two clades (Figure 3), one containing dicots and the other monocots (Poaceae). The dicots included *Arabidopsis thaliana*, *Brassica napus*, *Gossypium raimondii*, *Glycine max*, *Solanum tuberosum*, and *Beta vulgaris*, while the monocots contained *Triticum aestivum*, *Hordeum vulgare*, *Oryza sativa*, *Sorghum bicolor*, *Zea mays*, and *Setaria italica*. The clade of monocots was further divided into two clusters containing plants belonging to Pooideae (wheat, barley, and rice) and Panicoideae (sorghum, corn, and millet), respectively. Three genes of *Triticum aestivum* were revealed in one monophyletic group, while two genes of *Zea mays* and one gene of *Sorghum bicolor* formed another monophyletic group. In the clade of dicots, most species containing more than one *VHA-H* gene (*Gossypium raimondii*, *Glycine max*, and *Solanum tuberosum*) were revealed as monophyletic except for *Brassica napus*, which had six *VHA-H* genes with *Arabidopsis* embedded. The phylogenetic trees derived from Bayesian analysis (data now shown) showed largely congruent topologies to those revealed by the neighbor-joining tree.

### 2.3. Structures of the VHA-H Genes

We identified both the N-terminal and C-terminal domains and the intron phases of the *VHA-H* genes based on analyses of exon-intron structures (Figure 3). The coding region (CDS) of the *VHA-H* gene consisted almost entirely of the V-ATPase-H N-terminal and the V-ATPase-H C-terminal domains, besides the amino acid sequences at both ends of the CDS and the linker region connecting the two domains. The analysis of gene structures also showed similar exon-intron arrangements among most of the identified *VHA-H* genes. The gene YPR036W of *Saccharomyces cerevisiae* showed the simplest structure by having no introns. The positions of the C-terminal domains in the 12 species of plants were highly conserved and were all located on the last two exons of the *VHA-H* genes. However, the positions of N-terminal domains varied considerably in the 12 plants and were mapped on most of the exons. These results clearly showed that many alternative splicing sites were located on the N-terminal domain. The 22 identified *VHA-H* genes from 11 species of main crops ranged in size from 3301 bp (*BnVHA-H3*) to 8112 bp (*BnVHA-H5*), both corresponding to *Brassica napus*. The variations in size among different genes were mainly due to the number and length of introns. For example, the gene *BnVHA-H5* was 4144 bp in length and the length of its transcript was 2075 bp, while the gene of *BnVHA-H6* was 3313 bp in length and its transcript was 2049 bp in length. Both transcripts of the genes *BnVHA-H5* and *BnVHA-H6* were similar in length and they all had 10 introns, while the variation (813 bp) between the lengths of these two genes was due to intron length. The gene *GrVHA-H1* was 4522 bp in length with 10 introns, while *GrVHA-H2* was 5649 bp but with 11 introns and their transcripts were 1392 bp and 1446 bp in length, respectively. This showcases variation in the lengths of the genes caused by the number and lengths of introns. It is noteworthy that 5 of the 6 genes of *Brassica napus* shared similar lengths except for one (*BnVHA-H5*), which had almost twice the length of any of the other five genes from the same species, mainly due to one extremely long intron.

An analysis of phases of introns revealed that each gene contained the three phases known to disrupt (phases 1 and 2) and not disrupt (phase 0) codons (Figure 3). The largest proportion of intron phases of all the genes was phase 0 (60%), followed by phase 2 (34%) and phase 1 (6%). Both the N-terminal and the C-terminal domains in these 12 plants shared similar intron distribution patterns. For example, the phases of the first three introns in the N-terminal domain shared the same patterns (i.e., phase 0, phase 0, and phase 1) in all 12 species of plants, while the C-terminal domain ended with the sequence of phase 2, phase 0, phase 0, phase 0, phase 0, and phase 0, in that order. Furthermore, the intron distribution patterns of the different genes in the same species were also highly conserved.

### 2.4. Splice Variants of VHA-H Genes

Due to the existence of alternative splicing sites, a total of 7 genes in 6 species of crops contained multiple transcripts (Table 3 and Table 4). These transcripts generated a total of 26 putative translation products making up 63.45% of the total (41) putative translation products inferred from the 22 identified *VHA-H* genes in the 11 crop species. The alternative splicing events occurred mainly in the N-terminal domain, which is the main functional region of the enzyme. There was a total of 51 splicing sites, 27 of which derived from exon skipping, 9 from alternative 5′ splice sites, 6 from alternative 3′ splice sites, and 8 from mutually exclusive exon events (Table 3). The exon skipping events occurred on 5 out of the 7 genes containing multiple transcripts and on many exons. The alternative splicing sites occurring in the UTRs were identified in 6 out of the 7 genes and accounted for more than half of all splicing sites with some transcripts having only the alternative splicing in the UTR. On the one hand, more transcripts were revealed in species with fewer genes, e.g., barley had only one gene (*HvVHA-H*) but with six transcripts. On the other hand, species with more genes had fewer transcripts. For example, canola had 6 genes but with each gene having only one transcript. Furthermore, crops with wide planting areas had more than one gene and each had multiple splice variants. For example, maize had two genes (*ZmVHA-H1* and *ZmVHA-H2*) with each gene having three and five transcripts, respectively.

### 2.5. Analysis of Cis-acting Elements of VHA-H Promoters

**Figure 4:**
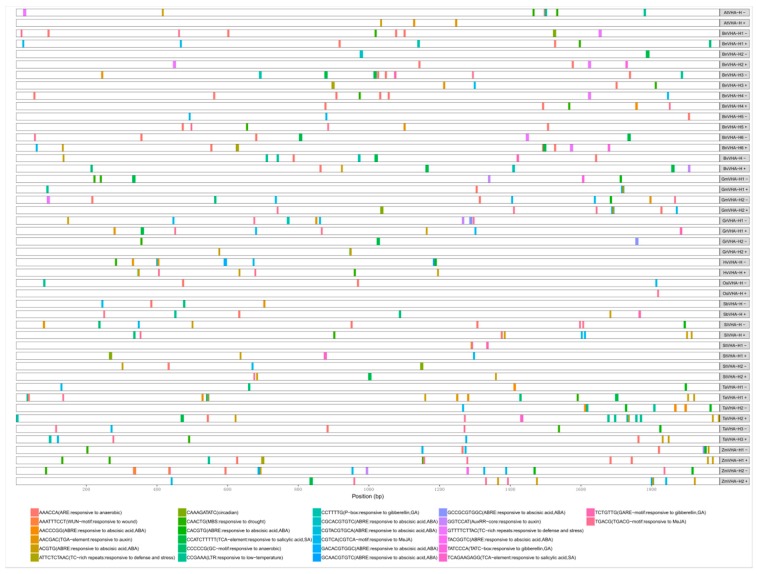
The cis-acting elements of the promoter regions that are 2000 bp upstream of the 23 *VHA-H* genes, with brief explanations of the functions of these elements.

The cis-acting elements of the promoter regions of the 23 *VHA-H* genes were analyzed (Figure 4). A total of 16 major cis-acting elements were revealed and categorized into three groups. The first group was involved in development, including circadian and AuxRR-core. The second group contained widely distributed phytohormone regulators such as P-box, TCA-element, GARE-motif, TGA-element, TATC-box, TGACG-motif, CGTCA-motif, and ABRE. The third group contained environmental stress-related elements which were abundantly present in the promoter regions, mainly LTR, WUN-motif, GC-motif, ARE, MBS, and TC-rich repeats. Among them, the most abundant elements were ABRE and ARE, which respond to hormones and stress, respectively.

### 2.6. Tissue-Specific Expression Patterns of VHA-H Genes

**Figure 5:**
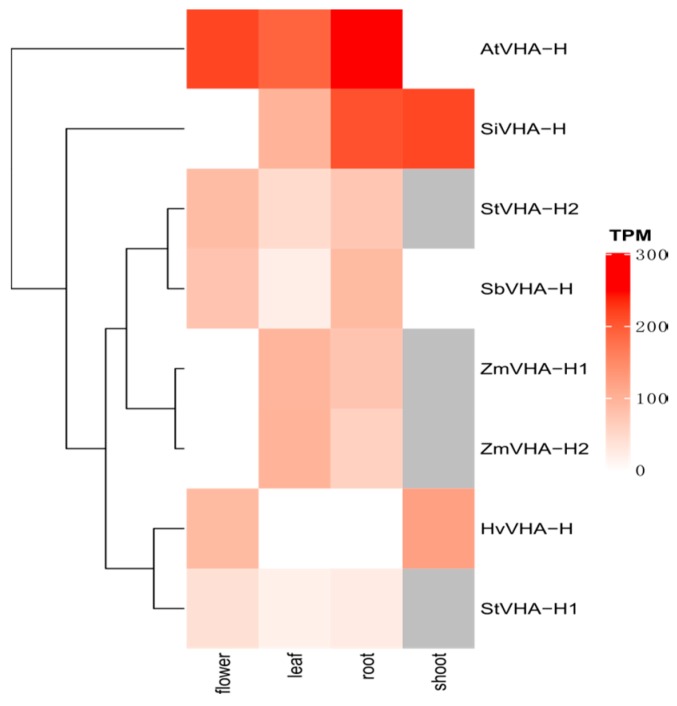
The expression patterns of *VHA-H* genes in different tissues. The heat level of each box represents the amount of expression, while the gray boxes indicate missing expression data. The clustering tree is on the left. TPM stands for transcripts per million.

Since the RNA-seq data of the *VHA-H* genes in 11 crops were incomplete in the *EnsemblPlants* database, we only obtained eight genes from six crop species to conduct the expression analysis. Most of these genes were highly expressed in roots and leaves, with more expressions in roots than in leaves except for those in maize (Figure 5). These *VHA-H* genes are evidently expressed higher in *Arabidopsis* and *Setaria italica* than in other species.

## 3. Discussion

Plant V-ATPase is a primary active proton pump of the endomembrane system [3]. It has multiple functions as a ‘house-keeping’ and stress response enzyme [27]. The *VHA-H* is a small subunit connecting the V_1_ and V_o_ complexes of the V-ATPase that is essential to the activity and stability of V-ATPase [22]. However, the *VHA-H* lacks genetic identification information in major crops and the evolutionary relationships of this gene family have not been investigated. Here we used well established bioinformatics and phylogenetic analysis methodology to identify and characterize the *VHA-H* genes of 11 main crops to illustrate both the structural diversity of the identified *VHA-H* genes and the proteomic diversity of the putative transcripts. These results will further our understanding of stress responses for crop improvement.

### 3.1. Identification of V-ATPase Subunit H Genes in the Main Crops

A total of 22 *VHA-H* genes were identified in 11 major crops with varied numbers of genes ranging from 1 to 6 among different plant species (Table 1). It was suggested previously that the number of genes encoding each of the V-ATPase subunits generally varied among different species of plants, suggesting the species-specific functions such as the acidification in the cell vacuole [10]. Furthermore, the injection of abscisic acid (ABA) significantly increased the citric acid content, accompanied simultaneously by the evident induction of the transcription levels of multiple subunits of the V-ATPase, including the subunit of H [10]. These results demonstrated that the *VHA-H* genes carry out specific functions in different species of plants. It is noteworthy that one *VHA-H* gene was identified in *Oryza sativa* of the Indica Group (Table 3) but no *VHA-H* gene was found in Japonica rice, indicating Indica rice had higher genetic diversity than Japonica rice. This observation agrees with previously reported results [28]. More importantly, the lengths of the ORFs, the amino acid sequences, the isoelectric points, and the number of exons of these 22 *VHA-H* genes were highly consistent among all species of plants (Table 1), indicating that members in the *VHA-H* gene family are evolutionarily highly conserved in the crop plants we surveyed. This conservation is presumably important and necessary for maintaining the specific functions of these genes across a large taxonomical spectrum.

### 3.2. Protein Sequence Alignments and Phylogenetic and Motif Analyses

Alignments of the amino acid sequences of the *VHA-H* genes showed that the sequence variations were in the middle portions of the N-terminal domain (Figure 1). These results are consistent with those of the gene structural analysis, which showed more variations in the N-terminal domain than the C-terminal domain (Figure 3). The conservative nature of the C-terminal domain was previously reported based on phylogenetic studies of the N-terminal and C-terminal domains of the V-ATPase proteolipid [29]. By using the N-terminal and C-terminal domains as separate entries for phylogenetic reconstruction, Gogarten [29] suggested that the front and back halves of the V-ATPase proteolipid were derived from a gene duplication that occurred after the bifurcation of the *Sulfolobus acidocaldarius* sequence and before the radiation of the eukaryotes. Furthermore, the results of the motif analysis showed clearly that the gains and losses of motifs all occurred in the N-terminal domain (Figure 2), suggesting again the more conservative nature of the C-terminal domain than the N-terminal domain of the V-ATPase. Our results revealed that among the 15 conserved motifs of the protein sequences putatively encoded by the *VHA-H* genes, motifs 1-14 were highly consistent in types, numbers, and distribution modes across main crops (except for *BnVHA-H3* and *BnVHA-H5* in *Brassica napus*), most notably sharing the same arrangement patterns. The *E*-values of motifs 1 and 2 were the highest among all of the 15 motifs. Since the C-terminal domain is mainly made up of both motifs 1 and 2, *E*-values again suggest that the C-terminal domain is more conserved than the N-terminal domain (Table 2, Figure 2). Overall, the observed positional changes of the motifs of all of the identified genes were consistent within the positions of both the N-terminal and the C-terminal domains (Figure 3). It is worth noting that the linker region of six amino acids connecting both the N-terminal and C-terminal domains was entirely included in motif 11, indicating that the upstream region of motif 11 belongs to the N-terminal domain (Figure 1, Figure 2).

The V-ATPase is an evolutionarily conserved and ancient enzyme with remarkably diverse eukaryotic functions [30]. Therefore, it has been speculated that phylogenetic analysis of the *VHA-H* gene family could help reconstruct evolutionary relationships among widely different organisms. In fact, the V-ATPase subunits have been used in phylogenetic analyses of various groups of organisms [9]. 

Our phylogenetic analysis of the *VHA-H* gene family showed the separation of the monocots and the dicots into distinct monophyletic groups harboring various clusters that were congruent with currently recognized taxonomic groupings (Figure 2). The phylogenetic relationships of gramineae also largely agree with those reported previously based on the evolutionary studies of the subunits of *VHA-H* genes in plants [9]. Furthermore, the evolutionary studies of the *VHA-H* genes suggested clear distinctions of various orders of insects (e.g., Diptera, Lepidoptera, and Orthoptera) [31]. Collectively, results suggest that the *VHA-H* gene is an evolutionarily ancient genetic marker that appeared before species diversification.

### 3.3. Gene Structural Diversity

The structural diversity generated by losses or gains of introns within gene families is one of many evolutionary mechanisms that promote variability [32]. However, intron positions and intron phases of the 12-oxo-phytodienoic acid reductase (OPR) genes were found to be well-conserved, with some introns being conserved in all plant lineages [33]. It has been speculated that lineage-specific intron loss events might have occurred during the expansion and structural evolution of OPR genes, which generated gene structural diversity [33,34]. Our results showed that the number and phases of introns were conserved in individual species while exhibiting inter-species diversity (Figure 3). For example, identical numbers and phases were observed in the introns of three species of plants, *Glycine max* (*GmVHA-H1* and *GmVHA-H2*), *Zea mays* (*ZmVHA-H1* and *ZmVHA-H2*), and *Triticum aestivum* (*TaVHA-H1*, *TaVHA-H2*, and *TaVHA-H3*). However, the numbers and phases of introns were different among different species of crops. For example, the fourth intron was phase 1 in *Zea mays*, phase 0 in *Triticum aestivum*, and phase 2 in *Arabidopsis* (Figure 3). It has been suggested that the presence of introns in the same positions in orthologous genes from distant eukaryotes may reflect evolutionary conservation rather than parallel gain [35]. Our analysis of phases of introns showed that the first 3 introns of the N-terminal domain and the last 5 introns of the gene exon/intron structure were conserved in all 11 species of crops, which shared the same patterns of phases. In contrast, the introns in the middle portion of the N-terminal domain were diverse, showing mostly phases 1 and 2 in various orders (Figure 3). Furthermore, introns with phase 0 were commonly located in more conserved areas, e.g., in the C-terminal domain (Figure 3). The C-terminal domain was more conserved and shorter than the N-terminal domain because many alternative splicing sites were located on the N-terminal domain (Figure 3). These results agree with those previously reported showing that introns with phase 0 were normally located in more conserved portions of genes than introns with phases 1 or 2 [36]. Previous studies demonstrated that conserved intron positions were found within a variety of ancient protein modules, suggesting that the initial function of exons did not represent the boundaries of functional protein modules, but that the domain itself was assembled from exons [37]. These results suggest that the phases of introns and the numbers of the *VHA-H* genes were well-conserved and that some introns (i.e., those located in the middle portion of the N-terminal domain) maintained their specificity.

Gene structural analysis also showed multiple *VHA-H* genes in some species, with individual genes showing varied gene structures. For example, both ends of the *VHA-H* gene in *Brassica napus* maintained equally conserved structures. However, the numbers and phases of the introns largely changed in the middle portion of the gene in comparison to other crops (Figure 3) and its genetic structure was rather diverse in the N-terminal domain, when compared to the C-terminal domain (Table 1, Figure 3). Our results revealed the diversity of both exons and introns of the N-terminal domain. Since the N-terminal domain is the major functional domain of the *VHA-H* gene product, the diversity of the *VHA-H* gene structure revealed in its N-terminal domain suggest the existence of functional diversity. Previous studies showed that single intron loss and gain contributed to the diversification of gene structure, and consequently, functional diversity and divergence, during the evolution of the NAD(H) kinase genes in plants [32]. Results of our analysis of gene structures indicated that the changes in the number and position of exons mainly occurred in the N-terminal domain, while the C-terminal domain was left largely unchanged (Figure 3). It was previously reported that the activation and functions of the V-ATPase complex do not require the first 179 amino acids of the N-terminal domain and the minimal region capable of activating the V-ATPase contains 174 amino acids at positions 180–353 of the N-terminal domain [25]. These results suggest that the changes in the N-terminal domain cause exon variations, leading to changes in functional specificity of the V-ATPase enzyme.

### 3.4. Splice Variants

Alternative splicing has been considered as one of the most important mechanisms contributing to the protein structural and functional diversity [36,38]. Alternative splicing is involved in many physiological processes of plants, including the response to biotic and abiotic stresses [39,40,41,42]. Specifically, the alternative splicing events were increased in *Arabidopsis* under salt stress probably due to an acclimation response [39]. In some cases, alternative splicing may alter the domain architecture of kinases, influence their subcellular localizations, and enhance the ability to cope with stress via transcriptomic plasticity [42]. Our studies showed that alternative splicing events occurred mainly in the N-terminal domain, which is the main functional region of the *VHA-H* gene product, showcasing higher variability relative to the C-terminal domain (Table 2). We also showed that the *VHA-H* genes in crops of wide planting area or with more than one family member contained multiple splice variants (Table 4). For example, there were two *VHA-H* genes (*ZmVHA-H1* and *ZmVHA-H2*) in *Zea mays* with each gene having three and five transcripts, respectively. Based on these results, we speculate that alternative splicing events occurring in the N-terminal domain, the functional unit of the enzymes, result in diverse functions needed to cope with varied environments.

Although the detailed functions of the alternative splicing variants in *VHA-H* genes have not been investigated in crops, studies in mouse and zebrafish suggested that two isoforms derived from gene *ATP6V1H* encoding the subunit H of V-ATPase were due to alternative splicing [16]. The amounts and the ratios of these two transcripts varied greatly among various types of tissues or cells suggesting that the selective expression of these two splice variants had different effects on the craniofacial development of zebrafish [16]. Our study of the 11 major crops showed that the transcripts of the *VHA-H* genes were diverse with some genes having multiple transcripts. For example, one *VHA-H* gene (*HvVHA-H*) of *Hordeum vulgare* had six transcripts, which is the largest number of transcripts uncovered in this study. It is also important to point out that more than half of the alternative splicing sites occurred in the UTRs (Table 3). The UTR sequences are known to play crucial roles in the post-transcriptional regulation of gene expression, including modulation of mRNA transport out of the nucleus [43], translational efficiency, and subcellular localization and stability [44]. Thus, alternative splicing of the UTRs could play an important role in the transcription process in the major crops we investigated. Furthermore, only the 5′ UTR but not the 3′ UTR in maize contained a cleavage site, while intron retention occurred in the 5′ UTR in the *ZmVHA-H2.4* transcript, making the 5′ UTR longer (Table 3). It has been reported that long and various 5′ UTRs provide more and different regulatory elements that might influence the efficiency of transcription, translation, and even the functions of the protein products of a single gene [45]. In wheat, only the 3′ UTR had alternative splicing sites, while alternative splicing occurred in both 3′ UTR and 5′ UTR in several other species (e.g., barley, millet, and potato, Table 3). It was previously demonstrated that both the 5′ and 3′ UTRs in pre-mRNAs play a variety of roles in controlling eukaryotic gene expression, including translational modulation [46]. For example, it was reported that both mRNA splicing and AU rich elements in the 3′ UTR can inter-dependently influence β-catenin mRNA stability [47]. These results suggest that the diversity of transcripts in major crops may lead to protein diversity, consequently, promoting the functional diversity of the *VHA-H* genes.

### 3.5. Cis-Acting Elements of the VHA-H Promoters

As an ancient eco-enzyme, V-ATPase plays an important role in plant development and adaptation [8]. Our results showed that the promoter elements of the *VHA-H* genes contained mainly the development and adaptation-related elements that respond to hormones and stress, respectively (Figure 4), suggesting that the *VHA-H* promoters play important roles in regulating plant responses to hormones and environmental stress. In our study, many hormones were associated with the *VHA-H* promoter elements, including abscisic acid (ABA), auxin, gibberellin (GA), methyl jasmonate (MeJA), and salicylic acid (SA) (Figure 4). Studies have shown that salt tolerance can be improved by GA and SA regulation [48,49], while cold tolerance can be improved by MeJA and ABA [50]. Furthermore, the ABA-responsive element (ABRE), which is the major cis-acting element for ABA-responsive gene expression, was the most abundant element of the *VHA-H* promoter regions (Figure 4). Studies have suggested that the control of the expression of ABA signaling factors may improve tolerance to environmental stresses [51]. For example, it was reported that ABA was associated with salt stress [52]. Similarly, NaCl-induced salt stress resulted in a significant accumulation of ABA in root tissues [53]. In addition, Shim et al. [54] reported that the content of SA increased in rice seedlings stressed by NaCl treatment. These studies suggest that there is a strong correlation between hormone regulation and environmental stress. This is supported by our study, which shows that both type and number of *VHA-H* promoter elements dominate responses to hormones and stress (Figure 4). These results suggest that the *VHA-H* promoters are associated with stress, suggesting that the *VHA-H* gene is both a housekeeping gene and a stress response (ecological) gene.

### 3.6. Tissue-Specific Expression Patterns of VHA-H Genes

Roots and leaves are generally important organs for plant growth and development. Studies showed that V-ATPase plays important roles in nutrient absorption and translocation and in the particular functions of guard cells [11,12], which are closely related to the functions of the roots and leaves. Our results showed that most of the *VHA-H* genes were expressed at high levels in roots and leaves (Figure 5), indicating that *VHA-H* genes may be important for the functions of these organs. We speculate that *Arabidopsis* maintains the features of wild plants and *Setaria italica* shows strong adaptability in various drought and barren growing environments. These features may be attributable to the higher expression of *VHA-H* genes relative to other crop species (Figure 5). Our results showed that the expression of *ZmVHA-H* and *SiVHA-H* genes in flowers was generally low, while high in *HvVHA-H*, *SbVHA-H*, and *AtVHA-H* (Figure 5). Since the flowers are one of the most important organs affecting seed yield, we further speculate that the *VHA-H* genes may be associated with yield in *Hordeum vulgare* and *Sorghum bicolor*.

## 4. Materials and Methods

### 4.1. Identification of VHA-H Genes

The protein sequences of the VHA-H gene family present in Gossypium raimondii, Oryza sativa Indica Group, Brassica napus, Beta vulgaris, Glycine max, Hordeum vulgare, Solanum tuberosum, Setaria italica, Sorghum bicolor, Triticum aestivum, and Zea mays were obtained from the EnsemblPlants database release 41 (Available online: http://plants.ensembl.org/info/website/ftp/index.html). BLASTp was used to identify the putative proteins encoded by the VHA-H genes from the 11 main crops using the protein sequence encoded by the VHA-H gene of Arabidopsis thaliana (AT3G42050, retrieved from EnsemblPlants database (Available online: http://plants.ensembl.org/index.html)) as query. A local protein database was created using BioEdit [55]. The Pfam database (Available online: http://pfam.xfam.org/) [56] was used to verify the predicted protein sequences of VHA-H genes and their corresponding two domains, i.e., V-ATPase-H-N (PF03224) and V-ATPase-H-C (PF11698). The VHA-H genes were named according to Shi et al. [10]. Characterizations of identified members of the VHA-H gene family, including accession numbers, chromosomal locations, ORF lengths, and numbers of exons and introns were retrieved from the EnsemblPlants database with one exception, i.e., the information of the chromosomal locations of canola was obtained from the Brassica napus Genome Resources (Available online: http://www.genoscope.cns.fr/brassicanapus/). Genes with incomplete gene sequences and domains were removed from further analyses. Basic physical and chemical parameters, i.e., molecular weight (MW) and theoretical isoelectric point (PI) of putative proteins of VHA-H genes, were calculated by using the compute pI/MW tool of the Expert Protein Analysis System (Available online: ExPAsy, https://web.expasy.org/compute_pi/).

### 4.2. Phylogenetic and Protein Motif Analyses

Amino acid sequences encoded by 24 *VHA-H* genes from 12 species of plants and yeast were aligned using ClustalW in MEGA-X [57]. Phylogenetic analysis of these 24 amino acid sequences was conducted by the neighbor-joining method with 1,000 bootstrap replicates using MEGA-X and by Bayesian inference using MrBayes [58] with the *ScVHA-H* sequence of yeast (*Saccharomyces cerevisiae*) as the outgroup. Conserved protein motifs of the 24 proteins putatively encoded by the *VHA-H* genes were identified with MEME (Available online: http://meme-suite.org/tools/meme) based on the full-length protein sequences of each putative member of the *VHA-H* gene family.

### 4.3. Gene Structure of VHA-H Genes

The Gene Structure Display Server (GSDS, available online: http://gsds.cbi.pku.edu.cn) was used to analyze the exon–intron structures within the coding sequences and the genomic sequences of each predicted *VHA-H* gene derived from the *EnsemblPlants* databases [59]. To illustrate the evolutionary patterns of introns, the phases of introns were selected when the structures of *VHA-H* genes were visualized with the tools at GSDS.

### 4.4. Splice Variants of VHA-H Genes

The sequences of splice variants of the 11 crops were retrieved from *EnsemblPlants*. Analysis of splice variants was conducted as previously described [60]. Specifically, all alternative splicing sites were classified into five types (i.e., exon skipping, mutually exclusive exons, alternative donor site, alternative acceptor site, and intron retention) based on [61]. The transcripts showing similar gene structure to that of *Arabidopsis thaliana* was selected as the wild type (WT), which was used as a reference for other gene transcripts to determine the types of alternative splicing.

### 4.5. Analysis of cis-acting Elements of VHA-H Promoters

The 2000 bp regions upstream of the transcription start site of all *VHA-H* genes in *Arabidopsis* and 11 crop species were obtained from *EnsemblPlants*. Cis-acting elements present in these upstream regions were predicted with the PlantCARE tool that is available online (Available online: http://bioinformatics.psb.ugent.be/webtools/plantcare/html/) [62,63].

### 4.6. Tissue-Specific Expression Patterns of VHA-H Genes

The RNA-seq data of *VHA-H* genes in flower, leaf, root, and shoot were obtained from *EnsemblPlants*. Gene expression abundances were visualized as heat maps.

## 5. Conclusions

We have identified 22 *VHA-H* genes in major crop plants. We have further studied the alignments and evolutionary relationships of the amino acid sequences, gene structures of exons and introns, and protein motif and alternative splice variants of these putative genes. We suggest the N-terminal domain is the major source of protein diversity and presumably the main functional region of species-specific adaptation, while the C-terminal domain is conserved and probably retains the original functions and characteristics of an ancient V-ATPase. The *VHA-H* gene family in plants shows genetic structure and transcript diversities mainly in the N-terminal domain, which presumably is the main source of functional diversity of these genes. Results of this study contribute further understanding of the structure, function, and evolution of the *VHA-H* genes and their important species-specific roles for crop adaptation and improvement.

## Figures and Tables

**Table 1:** Molecular characterization of 23 *VHA-H* genes in 12 species of plants.

Gene	Species	Ensembl ID	Chromosome: Locations	ORF Length (bp)	No. of Exon	Deduced Polypeptide		
						Length (aa)	MW (Da)	PI
*AtVHA-H*	*Arabidopsis thaliana*	AT3G42050	3: 14,229,669−14,233,441	1326	11	441	50284.42	6.58
*GrVHA-H1*	*Gossypium raimondii*	B456_003G056300	3: 9,065,281−9,069,803	1392	11	463	52652.25	7.55
*GrVHA-H2*		B456_004G256400	4: 59,286,789−59,291,565	1446	12	481	54453.38	6.48
*OsiVHA-H*	*Oryza sativa Indica Group*	BGIOSGA025913	7: 19,885,495−19,891,284	1488	12	495	56165.18	9.07
*BnVHA-H1*	*Brassica napus*	BnaA01g02950D	A1: 1,436,042−1,439,972	1359	11	452	51168.20	6.40
*BnVHA-H2*		BnaA03g50930D	A3: 26,436,023−26,439,323	1353	11	450	51179.39	6.25
*BnVHA-H3*		BnaA08g11340D	A8: 10,300,929−10,304,240	1407	13	468	54010.52	6.36
*BnVHA-H4*		BnaC01g04210D	C1: 2,192,503−2,196,646	1359	11	452	51151.19	6.58
*BnVHA-H5*		BnaC03g77060D	C3: 5,609,855−5,617,966	1380	14	459	52350.47	7.13
*BnVHA-H6*		BnaC07g44770D	C7: 43,161,725−43,165,037	1353	11	450	51191.44	6.25
*BvVHA-H*	*Beta vulgaris*	BVRB_4g074640	4: 3,654,561−3,661,113	1398	11	465	52980.14	7.07
*GmVHA-H1*	*Glycine max*	GLYMA_02G059800	2: 5,381,068−5,387,111	1347	11	448	51058.20	6.53
*GmVHA-H2*		GLYMA_16G142600	16: 30,131,160−30,136,817	1347	11	448	51004.12	6.48
*HvVHA-H*	*Hordeum vulgare*	HORVU2Hr1G042700	2H: 214,952,610−214,958,367	1359	11	452	51420.65	7.58
*StVHA-H1*	*Solanum tuberosum*	PGSC0003DMG400007911	12: 2,431,205−2,438,621	1365	11	454	51347.52	6.36
*StVHA-H2*		PGSC0003DMG401011206	7: 1,259,585−1,265,564	1365	11	454	51545.58	6.43
*SiVHA-H*	*Setaria italica*	SETIT_029790mg	II: 42,735,808−42,741,948	1377	12	458	52115.37	6.76
*SbVHA-H*	*Sorghum bicolor*	SORBI_3004G347600	4: 67,696,418−67,701,157	1452	12	483	55159.96	7.56
*TaVHA-H1*	*Triticum aestivum*	TraesCS2A02G212100	2A: 196,521,667−196,527,664	1359	11	452	51412.63	7.57
*TaVHA-H2*		TraesCS2B02G237200	2B: 237,967,455−237,974,014	1359	11	452	51382.58	7.98
*TaVHA-H3*		TraesCS2D02G218000	2D: 181,306,271−181,312,853	1359	11	452	51426.61	7.57
*ZmVHA-H1*	*Zea mays*	Zm00001d006565	2: 211,028,576−211,035,348	1359	11	452	51428.57	7.55
*ZmVHA-H2*		Zm00001d021721	7: 161,424,744−161,430,332	1359	11	452	51554.77	7.56

**Table 2:** The amino acid compositions of the 15 motifs of the 24 *VHA-H* genes revealed by MEME. The relative height of each letter (standing for an amino acid) is proportional to the relative entropy of the corresponding amino acid at the given position. Amino acids are listed in the descending order of frequencies from top to bottom within each position. Site Count represents the number of species with a motif detected.

Number	Motif Logo	*E*-value	Site Count	Number of aa
Motif 1	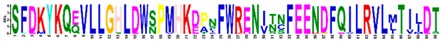	5.6 × 10^−1013^	23	50
Motif 2	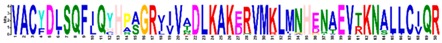	2.1 × 10^−981^	24	50
Motif 3	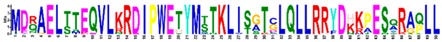	2.8 × 10^−889^	23	50
Motif 4	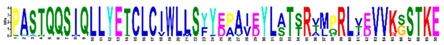	4.8 × 10^−939^	24	50
Motif 5	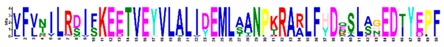	3.9 × 10^−886^	24	50
Motif 6	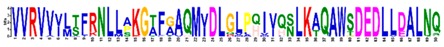	3.7 × 10^−823^	22	50
Motif 7	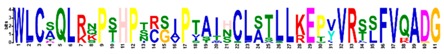	3.1 × 10^−669^	22	41
Motif 8	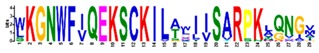	3.4 × 10^−475^	22	29
Motif 9	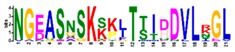	8.6 × 10^−260^	22	21
Motif 10	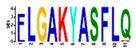	5.6 × 10^−179^	23	11
Motif 11	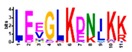	4.1 × 10^−116^	23	11
Motif 12	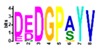	5.3 × 10^−105^	22	8
Motif 13	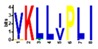	2.4 × 10^−081^	24	8
Motif 14	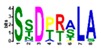	8.6 × 10^−061^	23	8
Motif 15	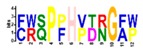	4.9 × 10^002^	2	12

**Table 3:** Splice variants of 7 *VHA-H* genes identified in 6 species of crops. Gene names are the same as those in Table 1. Alternative splicing sites occurring in the 5′ or 3′ UTRs are indicated in parentheses following the spliced exons.

Gene	Transcript	Ensembl Transcript ID	Predicted Amino Acid Length (aa)	Spliced Exon	Status
*GrVHA-H2*	GrVHA-H2.1	KJB26715	481		Wild type
	GrVHA-H2.2	KJB26714	351	Exon 1	Alternative 5′ donor site
				Exon 3	Alternative 3′ acceptor site
				Exons 4-7	Exon skipping
				Exon 12	Alternative 3′ acceptor site
*HvVHA-H*	HvVHA-H.1	HORVU2Hr1G042700.1	452		Wild type
	HvVHA-H.2	HORVU2Hr1G042700.2	452	Exon 1 (5′ UTR)	Alternative 5′ donor site
				Exon 12 (3′ UTR)	Mutually exclusive exons
	HvVHA-H.3	HORVU2Hr1G042700.3	452	Exon 1 (5′ UTR)	Alternative 5′ donor site
				Exon 12 (3′ UTR)	Mutually exclusive exons
	HvVHA-H.4	HORVU2Hr1G042700.4	450	Exon 1 (5′ UTR)	Alternative 5′ donor site
				Exon 11	Mutually exclusive exons
				Exon 12 (3′ UTR)	Exon skipping
	HvVHA-H.5	HORVU2Hr1G042700.5	494	Exon 1 (5′ UTR)	Alternative 5′ donor site
				Exon 10	Alternative 3′ acceptor site
				Exons 11-12 (3′ UTR)	Exon skipping
	HvVHA-H.6	HORVU2Hr1G042700.6	110	Exon 1 (5′ UTR)	Exon skipping
				Exons 2-7	Exon skipping
				Exons 10	Alternative 3′ acceptor site
				Exons 11-12 (3′ UTR)	Exon skipping
	HvVHA-H.7	HORVU2Hr1G042700.7	109	Exon 1 (5′ UTR)	Exon skipping
				Exons 2-7	Exon skipping
				Exon 11 (3′ UTR)	Mutually exclusive exons
				Exon 12 (3′ UTR)	Exon skipping
*StVHA-H2*	StVHA-H2.1	PGSC0003DMT400029149	454		Wild type
	StVHA-H2.2	PGSC0003DMT400029148	454	Exon 8	Mutually exclusive exons
				Exon 12 (3′ UTR)	Exon skipping
	StVHA-H2.3	PGSC0003DMT400029147	454	Exon 12 (3′ UTR)	Exon skipping
	StVHA-H2.4	PGSC0003DMT400029150	369	Exon 9 (3′ UTR)	Mutually exclusive exons
				Exons 10-11	Exon skipping
				Exons 12-13 (3′ UTR)	Exon skipping
	StVHA-H2.5	PGSC0003DMT400029145	144	Exons 1 (5′ UTR)	Exon skipping
				Exons 2-7	Exon skipping
				Exon 8	Alternative 5′ donor site
				Retained one exon between exons 8 and 9	Exon skipping
				Exon 11 (3′ UTR)	Alternative 3′ acceptor site
				Exon 12, 13 (3′ UTR)	Exon skipping
*SiVHA-H*	SiVHA-H.1	KQL26169	458		Wild type
	SiVHA-H.2	KQL26168	404	Exon 1 (5′ UTR)	Alternative 5′ donor site
				Exon 12 (3′ UTR)	Mutually exclusive exons
*TaVHA-H1*	TaVHA-H1.1	TraesCS2A02G212100.2	452		Wild type
	TaVHA-H1.2	TraesCS2A02G212100.1	455	Exon 12	Mutually exclusive exons
				Exon 13 (3′ UTR)	Exon skipping
*ZmVHA-H1*	ZmVHA-H1.1	Zm00001d006565_T002	452		Wild type
	ZmVHA-H1.2	Zm00001d006565_T001	379	Exons 4-6	Exon skipping
				Exon 7	Alternative 5′ donor site
	ZmVHA-H1.3	Zm00001d006565_T003	431	Exons 1-2 (5′ UTR)	Exon skipping
*ZmVHA-H2*	ZmVHA-H2.1	Zm00001d021721_T003	452		Wild type
	ZmVHA-H2.2	Zm00001d021721_T001	464	Retained two exons between exons 3 and 4	Exon skipping
				Exon 5	Exon skipping
	ZmVHA-H2.3	Zm00001d021721_T002	461	Retained two exons between exons 3 and 4	Exon skipping
				Exon 5	Exon skipping
				Exon 6	Alternative 3′ acceptor site
				Exon 7	Alternative 5′ donor site
	ZmVHA-H2.4	Zm00001d021721_T004	199	Exons 1-2 (5′ UTR)	Exon skipping
				Retained exon between exons 2 and 3 (5′ UTR)	Exon skipping
				Introns 3-5 (5′ UTR)	Intron retention
	ZmVHA-H2.5	Zm00001d021721_T005	431	Exons 1-2 (5′ UTR)	Exon skipping

**Table 4:** Number of transcripts per gene in 11 species of crops.

Species	*VHA-H1*	*VHA-H2*	*VHA-H3*	*VHA-H4*	*VHA-H5*	*VHA-H6*
*Gossypium raimondii*	1	2				
*Oryza sativa* (Indica Group)	1					
*Brassica napus*	1	1	1	1	1	1
*Beta vulgaris*	1					
*Glycine max*	1	1				
*Hordeum vulgare*	6					
*Solanum tuberosum*	1	4				
*Setaria italica*	2					
*Sorghum bicolor*	1					
*Triticum aestivum*	2	1	1			
*Zea mays*	3	5				

## Abbreviations

V-ATPase	vacuolar H^+^-ATPase
VHA-H	V-ATPase subunit H
UTRs	Untranslated regions
ABA	Abscisic acid
OPR	12-oxo-phytodienoic acid reductase
ORF	open reading frame
aa	amino acid
GA	gibberellin
SA	salicylic acid
MeJA	methyl jasmonate
